# Epigenetic stress memory in gymnosperms

**DOI:** 10.1093/plphys/kiae051

**Published:** 2024-01-31

**Authors:** Carl Gunnar Fossdal, Paal Krokene, Jorunn Elisabeth Olsen, Richard Strimbeck, Marcos Viejo, Igor Yakovlev, Melissa H Mageroy

**Affiliations:** Division of Plant Health and Biotechnology, Norwegian Institute of Bioeconomy Research, Ås 1431, Norway; Division of Plant Health and Biotechnology, Norwegian Institute of Bioeconomy Research, Ås 1431, Norway; Department of Plant Sciences, Faculty of Biosciences, Norwegian University of Life Sciences, Ås 1432, Norway; Department of Biology, Norwegian University of Science and Technology, Trondheim 7491, Norway; Department of Functional Biology, University of Santiago de Compostela, Santiago de Compostela 15782, Spain; Division of Plant Health and Biotechnology, Norwegian Institute of Bioeconomy Research, Ås 1431, Norway; Division of Plant Health and Biotechnology, Norwegian Institute of Bioeconomy Research, Ås 1431, Norway

## Abstract

Gymnosperms are long-lived, cone-bearing seed plants that include some of the most ancient extant plant species. These relict land plants have evolved to survive in habitats marked by chronic or episodic stress. Their ability to thrive in these environments is partly due to their phenotypic flexibility, and epigenetic regulation likely plays a crucial part in this plasticity. We review the current knowledge on abiotic and biotic stress memory in gymnosperms and the possible epigenetic mechanisms underlying long-term phenotypic adaptations. We also discuss recent technological improvements and new experimental possibilities that likely will advance our understanding of epigenetic regulation in these ancient and hard-to-study plants.

## Introduction

**Figure 1.**
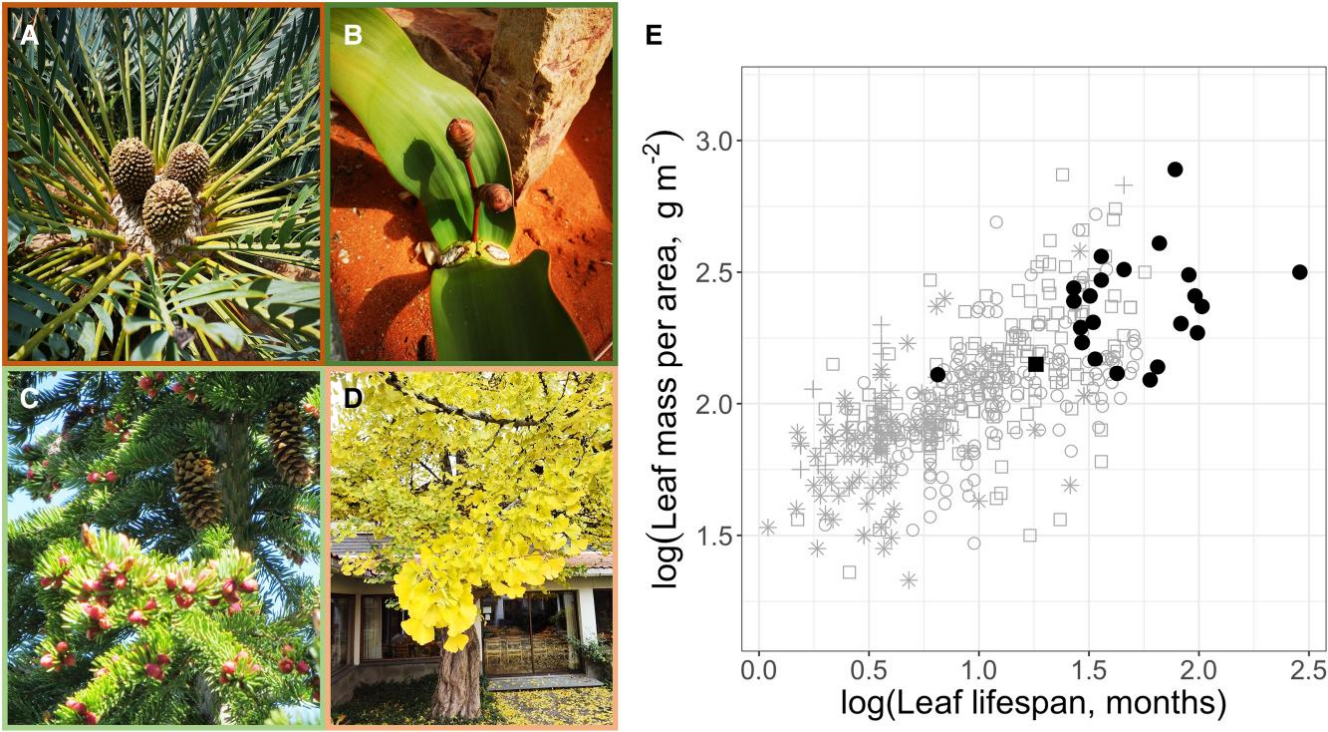
The gymnosperms include four divergent subclasses of land plants: **A)** Cycadidae (cycads), **B)** Gnetidae, **C)** Pinidae (conifers), and **D)** Ginkgoidae (ginkgo). Photos show a representative species from each subclass (all photos are by the authors). **E)** Conifers and other gymnosperms have long-lived, tough leaves with relatively low nitrogen concentrations and photosynthetic rates, reflecting their stress-adapted life histories. Leaf economic data for conifer (black symbols) and angiosperm (gray/open symbols) species in the Glopnet database (Wright et al. 2004). Circle = tree, square = shrub, star = herb, cross = graminoid.

Gymnosperms are long-lived, mostly evergreen woody plants with huge genomes. The gymnosperms comprise four highly divergent groups of land plants, which diverged from the lineage leading to angiosperms about 385 million years ago (Yang et al. 2022) (Fig. 1, A to D). The monotypic *Gingko biloba is* often described as a living fossil and became known to Westerners in the 17th century. Cycads, with about 370 species globally, are tropical and subtropical shrubs to small trees with palm-like leaves and cone-like reproductive structures. Conifers are mostly trees and a few shrubs in forest and woodland ecosystems from tropical to boreal and alpine regions, with over 700 species worldwide, including the world's tallest, greatest by volume, and oldest living trees. The fourth group, Gnetophytes, with about 70 species, is now recognized as a derived branch of conifers with some angiosperm-like characteristics, based on recent phylogenomic analysis (Yang et al. 2022). The deep evolutionary separation of gymnosperms from angiosperms suggests that these ancient genome giants may have evolved different epigenetic characteristics to angiosperms, which warrants further investigation (see Box 1 for a list of terms and definitions).

Box 1. **Terms and definitions.**

- **Canonical RNA-directed DNA methylation (RdDM):** a biological process in which 24 nt miRNAs, produced from double stranded-RNAs, guide the DNA methylation machinery to specific DNA sequences.
- **Constitutive defenses:** plant defenses that are present and ready to be used before attack or infection (preformed defenses).
- **Defense priming:** the sensitization of inducible defense responses for faster and/or stronger induction upon a subsequent challenge. Defense priming in conifers is thought to be governed by epigenetic mechanisms, but the details are still poorly understood.
- **Epigenetic memory:** a long-term reproducible/heritable change in gene expression or behavior that is induced by a previous stimulus (e.g. temperature, abiotic or biotic stress).
- **Epitype:** group of individuals with the same genome, but with different epigenomes resulting in different phenotypes. An example is “cool” and “warm” Norway spruce epitypes developing from embryos generated at different temperatures. These epitypes differ in phenotypic traits such as timing of bud break in the spring and bud set in the fall.
- **Euchromatin:** lightly packed chromatin (complex of nuclear DNA and protein), associated with active transcription.
- **Heterochromatin:** tightly packed chromatin (complex of nuclear DNA and protein), associated with transcriptional repression.
- **Inducible defenses:** plant defenses that are produced or activated only when an attack or infection occurs.
- **Linkage disequilibrium (LD):** the degree to which a gene in one location of the genome is inherited together with a gene at another location in the genome.
- **Methyl jasmonate (MeJA):** a volatile derivative of the plant hormone jasmonic acid that can act as an intra- and inter-plant defense signal.
- **Non-canonical RNA-direct DNA methylation:** alternative RdDM pathways that differ from the canonical RNA-directed DNA methylation in the size of sRNAs involved and in sRNA processing.
- **Phenotypic plasticity:** the ability of a single genotype to produce different phenotypes when exposed to different environmental conditions. The phenotypes of “cool” and “warm” Norway spruce epitypes are one example of phenotypic plasticity (see “Epitype” above).
- **Small RNAs (sRNAs):** a class of noncoding RNAs that are less than 200 nucleotides in length. They include microRNAs (miRNAs) and small interfering RNAs (siRNAs). sRNAs affect RNA stability and translation and are usually involved in RNA silencing.
- **Transposable elements (TEs):** repetitive, mobile DNA sequences that can replicate themselves within the genome. TEs are assigned to one of two classes based on their mode of replication: retrotransposons (copy-and-paste replication) and DNA transposons (cut-and-paste replication). TEs are typically highly methylated to prevent them from replicating by “jumping” in the genome.

Gymnosperms predominantly live and often dominate in stressful and somewhat stochastic environments, where the ability to acclimate to seasonal and long-term climatic trends and resist biotic challenges is key to individual survival. Conifer forests and woodlands cover most of the boreal and montane regions of the northern hemisphere, where growth is limited by short growing seasons, harsh winter conditions, and, in some regions, drought. Conifers also dominate temperate coastal rain forests and bog ecosystems in both hemispheres, where soils are leached by millennia of year-round rainfall and acidified by recalcitrant leaf litter or peat mosses, making for chronically nutrient-poor soils (Berendse and Scheffer 2009). In the tropics, conifers and cycads occupy nutrient-poor and leached soils, or xerophytic or high-elevation habitats (Goel and Khuraijam 2015). Many Gnetophytes are desert specialists, including, the ca. 56 species in the genus *Ephedra* and the bizarre, monotypic *Welwitschia mirabilis* of southern Africa (Fig. 1B).

A long life under chronic or episodic stress favors conservative growth and reproductive strategies, as reflected in gymnosperm leaf traits, which skew strongly toward the “live long and prosper” (as opposed to “live fast and die young”) end of the global leaf economic spectrum (Wright et al. 2004; Fig. 1E). Apart from *Ginkgo* and a few deciduous conifers, gymnosperms are evergreen, with long leaf lifespans, high leaf mass per area, low leaf nitrogen concentrations, and low mass-based photosynthetic rates (Reich et al. 1999; Zhang et al. 2015). To support long leaf lifespans, gymnosperm leaves are generally tough with thick cuticles and are defended by tannins and resins to conserve water and deter herbivores, resulting in recalcitrant leaf litter that promotes nutrient leaching and soil acidification.

During their long lifespans, gymnosperms are attacked by numerous insect pests and microbial pathogens, many of which have become invasive outside their natural range. Important insect pests include budworms (Lepidoptera), bark beetles (Coleoptera; Scolytinae), sawflies (Hymenoptera; Symphyta), and adelgids (Homoptera; Adelgidae), all of which may defoliate or kill entire stands of boreal and montane conifers, often following prior abiotic stress (Allison et al. 2023). These and other insect species may act as vectors for microbial pathogens. Insect-vectored and other fungal pathogens include rusts (*Cronartium* spp. and *Gymnosporangium* spp.), blights (*Diplodia* spp. and *Dothistroma septosporum*), bluestain fungi (Ophiostomatales, Microascales), and cankers (*Cytospora* spp.) (Asiegbu and Kovalchuck 2022). To face these biotic challenges, gymnosperms have evolved many constitutive and inducible defenses, such as production of specialized (secondary) metabolites and defensive enzymes (Krokene 2015; Anand et al. 2020; Liu et al. 2022; Yan et al. 2023). These defenses are executed over various timescales and tissues (Franceschi et al. 2005; Mageroy et al. 2023).

**Figure 2.**
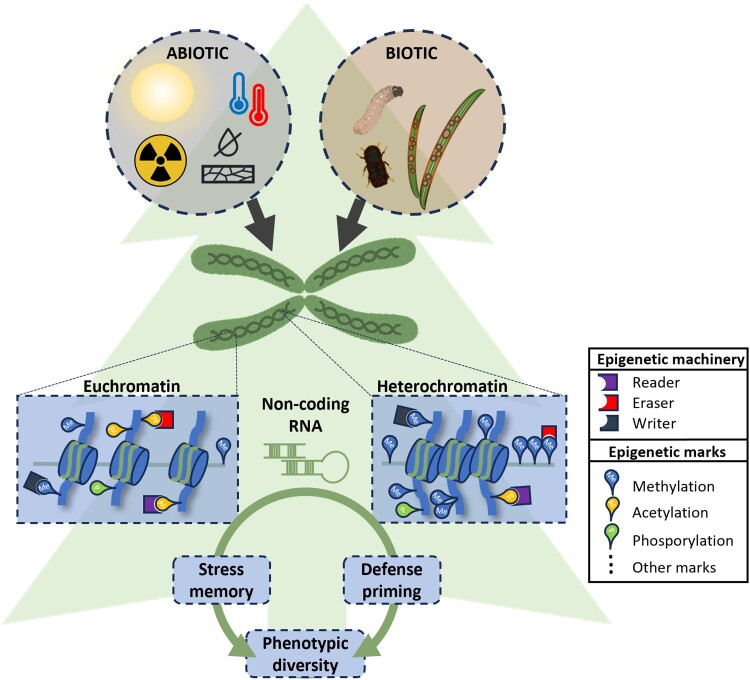
Epigenetic memory in gymnosperms. During their long lifetimes, gymnosperms face many and varied abiotic and biotic stresses. These may imprint epigenetic memories that improve the plants’ ability to adapt to stresses. The epigenetic machinery includes enzymes that makes DNA open (euchromatin) or closed (heterochromatin) for transcription, and thus regulate gene expression and ultimately the phenotype. Non-coding RNAs regulate gene expression by guiding RNA-directed DNA methylation to change a gene's methylation status or by post-transcriptional silencing of mRNAs. Most of our mechanistic knowledge about epigenetic memory comes from studies in angiosperms. Many epigenetic mechanisms are expected to be conserved across the plant kingdom, but there also appear to be some important differences between angiosperms and gymnosperms. See Box 2: Mechanisms of epigenetic memory for more details about epigenetic modifications in plants.

A major challenge for plants with very long generation times is to adapt to rapid environmental change through classical Mendelian inheritance. One solution is to have considerable phenotypic plasticity in key fitness traits. Epigenetic mechanisms, such as DNA methylation, histone modification, and sRNAs, allow for dynamic changes in gene expression and thereby phenotype (Wilkinson et al. 2019; Box 2) Epigenetic regulation of genes involved in gymnosperm stress tolerance likely plays an important role in the persistence of these remarkable plants (Fig. 2). Conifers are by far the largest and most studied group of gymnosperms and include important forest species worldwide that provide numerous ecosystem services, including timber production and climate change mitigation. Consequently, this review focuses on a few ecological and economically important conifers, mostly pine and spruce species, for which there is some knowledge about epigenetic stress memory. First, we will describe challenges with and reasons for studying epigenetic stress memory in conifers. Second, we present the current understanding of epigenetic memory to abiotic and biotic stress in conifers. Finally, we point out unresolved questions that require further research as well as recent developments in conifer genomic and epigenetic analyses that likely will enable a better mechanistic understanding of epigenetic memory in gymnosperms.

Box 2. **Mechanisms of epigenetic memory.**

The three main epigenetic mechanisms that modify gene expression are histone modification, DNA methylation, which is covalent and thus dynamic, and production of small RNAs (sRNAs).

- **Histone modifications** mainly include acetylation, methylation, and phosphorylation of histone proteins in eukaryote chromatin. These modifications are typically added to lysine (K) residues in histones 3 and 4 (H3 and H4). Ubiquitination, SUMOylation, glycosylation, and ADP-ribosylation are other possible but less common histone modifications. Chromatin immunoprecipitation sequencing (ChIP-Seq) is one method to identify histone modifications that occur in specific DNA regions. Antibodies for specific histone modifications are used to pull down DNA bound to these histones and the isolated DNA is then sequenced. Identification of genome regions with enriched sequencing reads (peak-calling) is then used to identify differentially modified histones.
- **DNA methylation** occurs on cytosine (C) nucleotide bases, although adenine also can be methylated to some extent. In plants, DNA methylation occurs in three different sequence contexts: CG, CHG, and CHH (where G represents guanine and H represents any base except for guanine). Increased methylation (hypermethylation) is typically associated with transcriptional repression, and reduced methylation (hypomethylation) with active transcription. In addition to methylation, cytosine may be modified by hydroxymethylation, formylation, or carboxylation, but the effects of these modifications are not well understood. Whole-genome bisulfite sequencing is a common technique used to determine where changes in DNA methylation occur. DNA is treated with sodium bisulfite to convert unmethylated cytosines into uracil. Untreated and treated DNA is then sequenced to identify methylated cytosines. Recently, long-read sequencing techniques, such as Nanopore and PacBio, have made it possible to detect DNA methylation without bisulfite conversion.
- **sRNAs** may act as guides for the DNA methylation machinery, allowing for precise targeting of specific “writer” enzymes to particular loci and other chromatin regions. sRNAs play important roles in the RNA-directed DNA methylation (RdDM) pathway, which is unique to plants. sRNAs may also be involved in post-transcriptional gene silencing by interfering with mRNA.

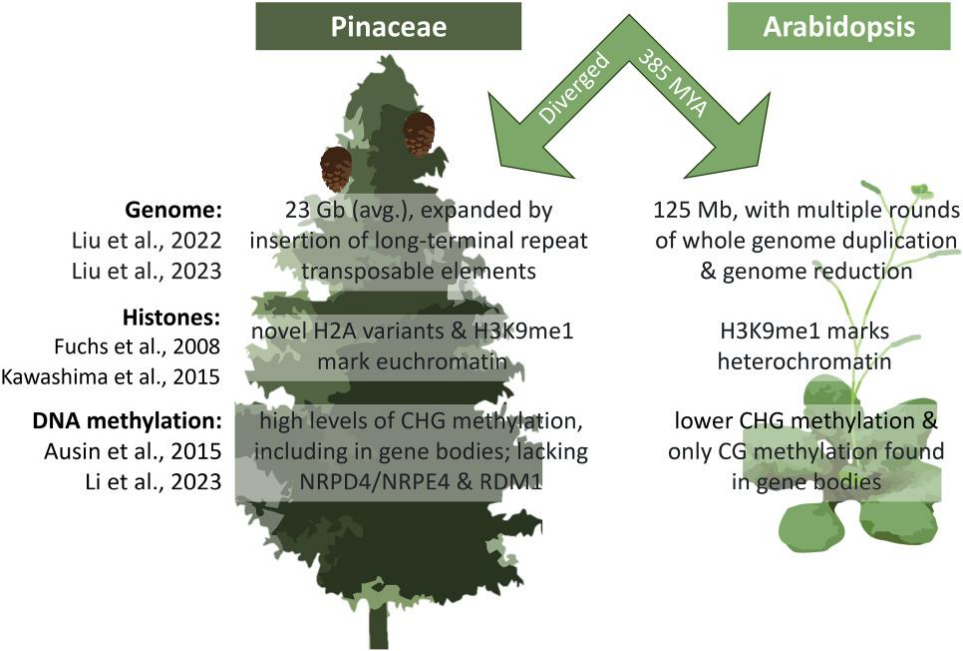

**Box 2 Figure.** Comparison of key life-history parameters and epigenetic mechanisms in gymnosperms (exemplified with conifers in the pine family) and angiosperms (the model plant *Arabidopsis thaliana*).

### Challenges with studying epigenetics in gymnosperms

Epigenetic studies in gymnosperms are challenging both due to certain unique characteristics of gymnosperms and the inherent complexity of epigenetic studies (see Box 2: Mechanisms of epigenetic memory). Specific challenges presented by working with conifers are long generation times for all but a few species, very large and complex genomes (up to 37 Gbp), the general lack of complete and accurate reference genomes, the complexity of data analysis, and the deep evolutionary divergence between gymnosperms and angiosperms.

Gymnosperms live for decades to millennia and have generation times of 20 years or more. Thus, it is challenging to study epigenetic changes across multiple generations to understand inheritance patterns. Additionally, at least in conifers, most phenotypic traits are polygenic with high and long-range linkage disequilibrium (LD) levels (Pavy et al. 2012; Larsson et al. 2013; Lu et al. 2016). This makes it difficult to obtain distinct mutants or even clones with defined phenotypic differences. Furthermore, some conifers show severe inbreeding depression that results in high mortality of selfed seeds, making it difficult to increase the zygosity in these outbreeding species (Williams and Savolainen 1996).

The specialized defensive metabolites that gymnosperms produce, as well as their wood chemistry, can make simple tasks such as extracting RNA and DNA challenging or impossible using conventional extraction kits. Once nucleotides are extracted, downstream analysis is often not straightforward. Gymnosperm genomes tend to be extremely large and complex (Soltis and Soltis 2013; Neale and Wheeler 2019; Gagalova et al. 2022; Shalev et al. 2022), with a very high proportion of repetitive sequences (ca. 75%) (Nystedt et al. 2013; Liu et al. 2022). Additionally, the gene space can be extensive, as average intron size ranges from 1 to 10 kbp (Nystedt et al. 2013; Warren et al. 2015; Liu et al. 2022; Niu et al. 2022). Thus, great sequencing depth and long reads are required to correctly assemble genomes. This, in turn, demands large data storage and computing capacity to analyze the data. The complexity involved in sequencing and analyzing gymnosperm genomes means that there still are few genome assemblies that can be used as reference genomes. Without a good reference genome, it is challenging to accurately map and interpret epigenetic modifications. Until now, the genome of only one conifer, Chinese pine (*Pinus tabuliformis*), has been assembled at the chromosome level and can be used as a high-quality reference for *Pinus* species (Niu et al. 2022).

Other challenges facing epigenetic studies of gymnosperms stem from the inherently complex nature of epigenetic modifications (Fig. 2; Box 2). Epigenetic marks often vary among individuals of the same species, within different tissues of the same individual, and even within the same tissues at different stages of development and time of year. Capturing this variability and understanding its functional significance in long-lived plants becomes very complex and requires careful consideration of which tissues to analyze and when to sample. Another challenge is that environmental factors can influence epigenetic modifications and therefore should be accounted for when designing experiments and interpreting results. Furthermore, epigenetic modifications may be interconnected and influence each other (Fang et al. 2022). Finally, epigenetic tools and resources that have been developed for model plant species such as *Arabidopsis thaliana* may not be directly applicable to gymnosperms, due to differences in genetics and epigenetic regulation. At present, this limits the number of available tools for studying epigenetics in gymnosperms.

### The importance of studying epigenetic stress memory in gymnosperms

Given the challenges and slow rate of progression in building knowledge about epigenetic stress memory in gymnosperms, one may ask why to pursue such studies at all. As described above, the ecology and evolutionary history of gymnosperms set them apart from model angiosperms. Unlike angiosperms, shifts in gymnosperm diversification have been more associated with evolution to increase climatic occupancy than by whole-genome duplication events (Stull et al. 2021; Gagalova et al. 2022). Although we expect many epigenetic mechanisms to be conserved across the plant kingdom, there also appear to be some important differences. Perhaps the most obvious difference between angiosperms and gymnosperms is their mode of reproduction. Angiosperms undergo double fertilization, which results in two fertilized tissues: a diploid embryo and a triploid endosperm (Mosher and Melnyk 2010). Gymnosperms have a single fertilization, which results in a diploid embryo and haploid endosperm with genes from the mother only. Angiosperms also possess seeds enclosed in a fruit, unlike gymnosperms, which have naked seeds. It is unknown if and how differences in the fertilization process and the divergent endosperm of angiosperms and gymnosperms impact the epigenetic state of the embryos- or trans-generational epigenetic memory (Chow and Mosher 2023).

Another notable difference between conifers and model angiosperms is the distribution of 24 nt microRNAs (miRNAs) (Chávez Montes et al. 2014). In angiosperms, highly abundant 24 nt miRNAs guide de novo DNA methylation through the canonical RNA-directed DNA methylation (RdDM) pathway (Cuerda-Gil and Slotkin 2016) (Box 1). This pathway plays an important role in regulating DNA methylation states and silencing transposable elements (TEs). In conifers, 24 nt miRNAs are restricted primarily to reproductive tissues, whereas 21 nt miRNA predominate in all other tissues (Nystedt et al. 2013; Niu et al. 2015).

Most genes involved in DNA methylation are conserved between all seed plants, but there are some notable absences in gymnosperms, such as a lack of DNA-DIRECTED RNA POLYMERASE IV SUBUNIT (*NRPD4*/*NRPE4*) and RNA-DIRECTED DNA METHYLATION 1 (*RDM1*) (Ausin et al. 2016; Li et al. 2023). Additionally, in conifers the largest subunit of RNA polymerase V (NRPE1) lacks both the argonaute (AGO) hook (which binds AGO proteins) and defective chloroplasts and leaves (DeCLs) motifs (a domain of unknown function) (Matzke and Mosher 2014). These dissimilarities in key epigenetic components raise questions about the role and mechanisms of RdDM in gymnosperms and suggest that a non-conical RdRM pathway may be the prevalent mode of de novo methylation in gymnosperm vegetative tissues (Cuerda-Gil and Slotkin 2016; Li et al. 2023).

Epigenetic effects of histone modifications (Fig. 2; Box 2) may also differ between gymnosperms and angiosperms. For example, methylation of lysine 9 on histone H3 (H3K9me1), which marks heterochromatin in angiosperms, was found to label euchromatin in Scots pine (*Pinus sylvestris*) and Norway spruce (*Picea abies*) (Fuchs et al. 2008). Additionally, Norway spruce has many variants of histone H2A, including some that are shared with other non-flowering vascular plants, some that are shared with flowering plants, and some that are unique to spruce (Kawashima et al. 2015). The possible roles of all these histone variants in epigenetic regulation are still unknown (Osakabe and Molaro 2023).

The unique features of the epigenetic machinery in gymnosperms illustrate why it is important to unravel epigenetic stress memory in these ancient seed plants, both to learn more about the diversity of epigenetic mechanisms across the plant kingdom and to understand how epigenetics contribute to the great adaptive flexibility and stress resilience of gymnosperms.

## Epigenetic memory induced by climatic and abiotic factors

### Warming temperature impacts during embryogenesis

**Figure 3.**
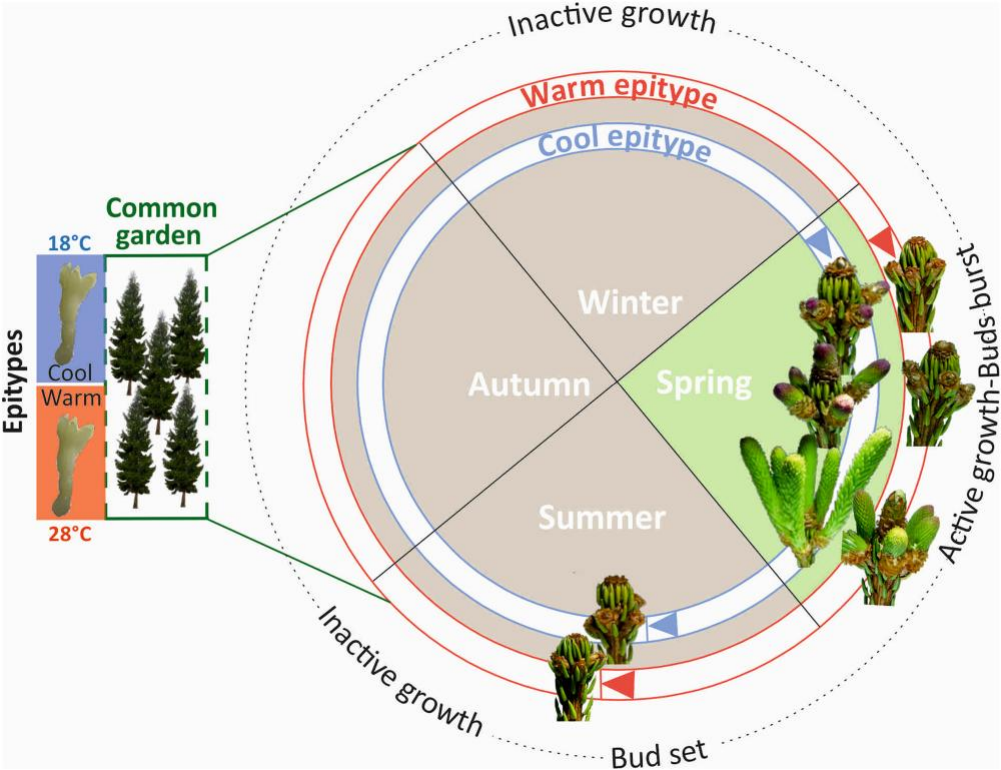
Climatic memory in Norway spruce. Bud phenology is depicted throughout the yearly cycle (summer, autumn, winter, and spring), with a focus on the epigenetically altered timing of bud burst between cool and warm epitype trees. Exposure of Norway spruce embryos to different temperatures (18 and 28 °C) induces lasting epigenetic change that affects bud phenology. When the resulting “cool” and “warm” epitype trees grow together under identical natural conditions (“common garden”), they have significantly different timing of bud burst in the spring, even though the epitypes are genetically identical. Bud swelling, bud burst, and shoot elongation start significantly earlier in the spring and early summer in the cool vs the warm epitype (“active growth”). These phenological differences have so far persisted for 20 years in the field. The new buds that are set in summer (“bud set”) are visually indistinguishable in the two epitypes for the rest of the yearly cycle (“inactive growth”).

Many boreal and temperate conifer species have photoperiodic and latitudinal ecotypes. The critical photoperiod required to sustain growth increases with latitude and results in a clinical (continuous) variation in the timing of winter bud development and other phenological traits (Olsen 2010; Olsen and Lee 2011). Traditionally, allelic variation was thought to be the only source of variation in the adaptive capacity of such ecotypes. However, more recently it has been shown that much of the variability in bud phenology between ecotypes can be explained by a memory of the temperature sum experienced during embryogenesis (Kvaalen and Johnsen 2008; Viejo et al. 2023) (Fig. 3).

Studies in Norway spruce indicate that an epigenetic memory of the photoperiod and temperature sum experienced during embryogenesis and seed development can influence bud phenology and frost tolerance in the progeny (Bjørnstad 1981; Johnsen and Skrøppa 1996; Johnsen et al. 2005a; Yakovlev et al. 2012; Solvin and Steffenrem 2019; Gömöry et al. 2020; Yakovlev et al. 2020; Viejo et al. 2023). This epigenetic memory increases phenotypic plasticity in climatic adaptation. When Norway spruce is exposed to increasing temperatures from 18 to 28 °C during zygotic or somatic embryogenesis, the resulting epitype trees show increasingly delayed bud set, bud burst, and cold de-acclimation in the spring (Johnsen et al. 2005a, b; Kvaalen and Johnsen 2008; Carneros et al. 2017) (Fig. 3). These altered phenotypes have been shown to persist for at least 20 years (Skrøppa 2022; Viejo et al. 2023). Similar epigenetic effects have also been observed in other conifer species (Dormling and Johnsen 1992; Greenwood and Hutchison 1996; Stoehr et al. 1998; Wei et al. 2001; Schmidtling and Hipkins 2004; Webber et al. 2005; Gömöry et al. 2020; Bose et al. 2020b).

Establishment of a temperature-induced epigenetic memory in Norway spruce is associated with changes in the DNA methylome and transcriptome reprogramming at both the mRNA and sRNA level (Yakovlev et al. 2012, 2020; Viejo et al. 2023). This includes a novel class of 32 nt sRNAs identified in embryos (Yakovlev and Fossdal 2017). Genes related to the epigenetic machinery, circadian clock, and phenology were differentially DNA methylated between epitypes. More details about the Norway spruce model system for epigenetic memory in gymnosperms are presented in Box 3: Epigenetic memory in conifers: the Norway spruce example.

Box 3. **Epigenetic memory in conifers: the Norway spruce example.**

**Background:** Norway spruce (*Picea abies*) was the first conifer species where epigenetic memory was studied intensively. Climatic memory effects were initially observed in grafts of selected plus trees that had been translocated from northern and high-altitude localities to seed orchards at more southern latitudes for improved seed production. Surprisingly, seedlings from seed lots produced in southern orchards differed considerably in certain phenotypic traits from comparable plants from natural stands in their area of origin. This included differences in important adaptive traits such as timing of terminal bud set and development of frost hardiness in the autumn (Bjørnstad 1981).

**A temperature memory:** Careful follow-up studies showed that an increase in the temperature sum experienced during embryogenesis induced a consistent change (delay) in bud phenology (see figure below). Together with associated changes in frost tolerance, this indicated an epigenetic shift in the annual growth cycle between epitypes (Johnsen et al. 2005b; Yakovlev et al. 2012, 2020; Viejo et al. 2023). This epigenetic memory is consistent with the idea that any one genome can give rise to many epigenomes. These epigenomes alter gene expression and increase phenotypic diversity and plasticity much faster than classical Mendelian selection and adaptation can do. The epigenetic memory induced during embryogenesis appears to be life-lasting and does not attenuate with time, unlike the more short-lived memory observed for defense priming in saplings and trees in response to stress, pathogens, wounding, or methyl jasmonate treatment. Temperature epitypes growing in the field are now reaching maturity, and seeds have been collected to determine if the temperature memory is trans-generational (see Advances Box).

**Epigenetic mechanisms:** The climatic memory in Norway spruce is associated with massive DNA methylation changes and reprogramming of the mRNA- and sRNA-omes (Yakovlev et al. 2012, 2020; Viejo et al. 2023). Key genes involved in the epigenetic machinery, phenology, and circadian clock pathways are differentially methylated. Notably, differential methylation is observed for key genes encoding DNA methyltransferases and ARGONAUTEs, as well as *FLOWERING LOCUS T-LIKE* (*FTL*)-genes involved in bud phenology (Viejo et al. 2023).

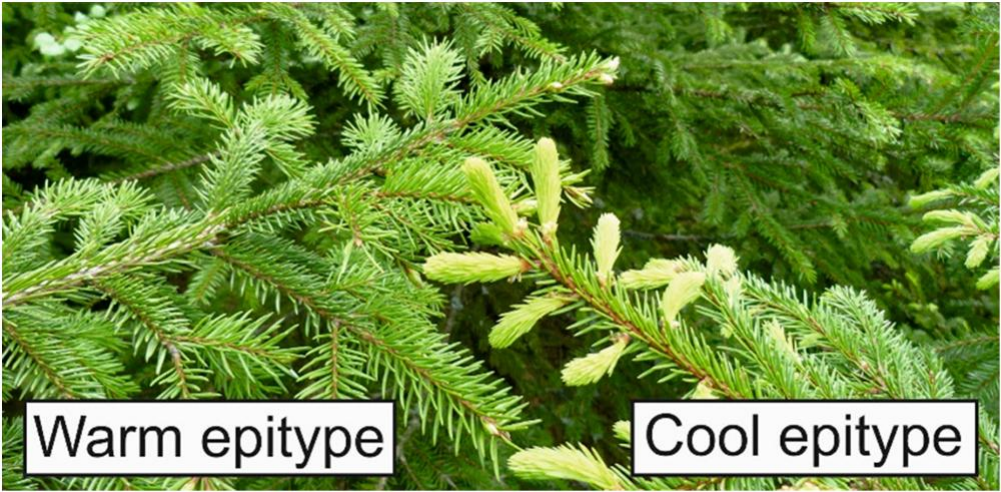

**Box 3 Figure.** Norway spruce epitypes originating from somatic embryos exposed to 18 °C (cool) or 28 °C (warm) during embryogenesis differ substantially in the timing of bud burst under common garden conditions.

### Low temperature

In the taiga forests of Siberia and Canada, winter minimum temperatures fall below −60 °C (Latysheva et al. 2007), with summer maxima above 35 °C and increasing due to climate change (Overland and Wang 2021). The aboveground tissues of conifers and other trees in these regions must acclimate and de-acclimate rapidly to take full advantage of the limited growing season, while minimizing risk of frost stress and injury. Boreal trees have incredible cold tolerance. Fully acclimated needle, bud, and bark tissues of boreal evergreen and deciduous trees will survive immersion in liquid nitrogen (N) (−196 °C), provided they are first slowly cooled to an intermediate temperature (typically −20 to −30 °C) (Sakai 1960; Strimbeck et al. 2007).

Frost acclimation and dormancy development is initiated by changes in day length and light quality in late summer and further driven by chilling temperatures in early autumn (Christersson 1978). Needles of taiga conifers attain liquid N tolerance by mid-October even if they are grown in an arboretum under a relatively mild autumn and winter climate (Strimbeck et al. 2008), indicating a strong genetic component in the timing and extent of frost hardening. De-acclimation in temperate and boreal trees is generally driven by heat sum accumulation above a threshold temperature (Morin et al. 2009; Rossi and Isabel 2017). Heat sum-driven de-acclimation allows for some flexibility in the timing but may result in precocious de-acclimation if there are warm periods during winter (Strimbeck et al. 1995).

Biochemical changes during frost acclimation include accumulation of oligosaccharides and compatible solutes (Angelcheva et al. 2014), changes in membrane lipid composition (Senser 1982), and accumulation of dehydrins (Kjellsen et al. 2013) and other late embryogenesis abundant (LEA) or other protective proteins. These changes involve expression of hundreds of cold-regulated (COR) genes. The photoperiodic component of dormancy development and early frost acclimation in trees involves circadian clock genes *FLOWERING LOCUS T* (*FT*)-, FT-TERMINAL FLOWER 1-*LIKE* genes (*FTL*), and *FLOWERING LOCUS C* (*FLC*)-like genes (Gyllenstrand et al. 2007; Olsen 2010). Although these pathways are best characterized in *A. thaliana* and other model species, homologs have been identified in some conifers (Chang et al. 2021).

Epigenetic modification can potentially fine-tune the timing of acclimation and de-acclimation to accommodate year-to-year variability and longer-term trends that occur within the long individual lifespans of coniferous species. In Norway spruce, expression of dehydrins (*DHNs*) associated with frost tolerance (Strimbeck et al. 2015) is impacted by epigenetic memory (Carneros et al. 2017). DHN transcript levels decrease gradually prior to flushing, a time when trees are very sensitive to frost. Epitypes that flush earlier show differential expression of these genes and the timing of decreased *DHN* expression is shifted, indicating that timing of bud burst-related gene expression, bud burst, and frost tolerance is epigenetically regulated in Norway spruce (Carneros et al. 2017).

Low temperature can serve as important signals for plant development, and it has epigenetic effects on seeds and induces flowering. Some angiosperms require seed stratification by exposure to low temperatures for efficient germination. Epigenetic mechanisms may be controlling developmental switches during seed growth and germination (Luján-Soto and Dinkova 2021). In gymnosperms, classical stratification by low temperature treatment appears to have some effect, but dry storage of spruce seeds at low temperatures does not improve germination (Cram 1951). However, cold stratification tends to increase germination in pine and spruce over that of just soaking the seeds in water (Houšková et al. 2021). Epigenetic mechanisms involved in stratification for gymnosperm seeds have not been studied in any detail. Chilling is another important temperature signal for plants. In angiosperms, the competence to flower can be epigenetically controlled by vernalization (Chouard 1960; Andrés and Coupland 2012), the induction of a plant's ability to flower by exposure to winter or an equivalent chilling treatment. In winter-annual ecotypes of *Arabidopsis*, high FLC expression prevents transition to flowering in nonvernalized plants (Michaels and Amasino 2000). In response to prolonged periods of chilling, a battery of chromatin modifiers, including polycomb group factors and histone acetyltransferases, methyltransferases, and deacetylases, modify chromatin at the FLC locus to repress FLC expression (Bouché et al. 2017). This cold-induced FLC epigenetic silencing is maintained in the plant and ensures floral induction under the longer days in late spring (Bouché et al. 2017). Since gymnosperms do not flower, the classical definition of vernalization does not apply to them, and there are no indications that chilling impacts male and female cone induction (Ma et al. 2022).

### Heat stress

Both angiosperms and gymnosperms possess heat stress memory, but the duration of this memory and to what extent it can be trans-generationally inherited is not known (Perrella et al. 2022). Studies in conifers show that moderate heat stress, where plants are exposed to 40 °C for 5 to 6 h, induces large transcriptomic and metabolomic changes at the whole-plant level (Escandón et al. 2017; Roces et al. 2022). Heat stress induces epigenetic reprogramming and transmission in the form of DNA methylation and histone modification marks, but the longevity of these changes is largely uncharted at the molecular and mechanistic level. Changes in miRNAs and accumulation of histone H2A are implicated in heat memory in Monterey pine (*Pinus radiata*) (Lamelas et al. 2020, 2022).

Exposure to heat stress during somatic embryogenesis seems to induce changes in DNA methylation, stress-related gene expression, and physiological traits in the resulting plants, such as net photosynthesis rates or stomatal conductance and transpiration (Do Nascimento et al. 2020, 2022; Castander-Olarieta et al. 2021). However, the plants were not followed long enough to characterize the persistence of heat stress memory over their life span. Another experiment (Pérez-Oliver et al. 2023) in maritime pine (*Pinus pinaster*) showed that the heat stress memory was retained even if female gametes were exposed to heat shock prior to somatic embryogenesis. Exposure to contrasting temperatures during somatic embryo maturation had a counterintuitive effect on heat stress resistance of the generated plants, as plants from embryos that matured at 18 °C were more resistant to high temperatures than plants from embryos that matured at 23 or 28 °C (Sales et al. 2022).

### Drought

Many conifers thrive in dry and drought-prone environments, notably at higher elevations in areas in the inland western United States and northwestern Mexico that support a high diversity of conifer species (Neale and Wheeler 2019). While drought stress is usually associated with high temperatures and heat stress, frost stress also results in cellular dehydration due to extracellular freezing of water. Because drought and frost stress have similar effects at the cellular level, conifers tend to have similar and overlapping tolerance mechanisms to both types of stress (McCulloh et al. 2023). Epigenetic adaptation to long-term drought could play a role in ensuring survival of species such as bristlecone pine (*Pinus longaeva*), which perseveres for millennia at high elevations in the desert mountains of the southwestern United States.

Some conifers are very sensitive to drought (Song et al. 2019). Drought-induced tree mortality is mostly associated with a low capacity to recover after periods of water limitation (García-García et al. 2022). There are indications of a drought stress memory in conifers, as Scots pine trees that have experienced more frequent, mild drought are less susceptible to subsequent extreme drought (Bose et al. 2020a). Additionally, there is some evidence that this memory is trans-generational (Bose et al. 2020b). However, epigenetic mechanisms of drought memory remain largely unknown in conifers (García-García et al. 2022).

### Ultraviolet and gamma radiation

Epigenetic memory of ultraviolet (UV) radiation likely occurs in conifers (Müller-Xing et al. 2014; Crisp et al. 2016). Evidence supporting a light stress memory in conifers is that UV-B-irradiated Norway spruce seedlings show a decrease in CG-DNA methylation and a concomitant increase in emission of specific terpenoids involved in stress protection (Ohlsson et al. 2013). Thus, an association between UV-B-induced defense and epigenetic regulation has been hypothesized (Ohlsson et al. 2013). Additionally, transcripts involved in epigenetic regulation have been shown to increase in Monterey pine after UV irradiation (Valledor et al. 2012), including the *MURASHI RNA BINDING PROTEIN 1* (*MSI1*), known to be involved in chromatin dynamics and epigenetic inheritance during mitosis, and *S-ADENOSYLMETHIONINE SYNTHASE 4* (*SHMS4*), which supplies the substrate for DNA methylation (Köhler et al. 2003; Ulrey et al. 2005).

Laboratory experiments and accidental exposure in the field, such as after the Chernobyl nuclear power plant accident in April 1986, have revealed that plants differ in their sensitivity to ionizing radiation (Caplin and Willey 2018; Blagojevic et al. 2019). Conifers, such as Scots pine and Norway spruce, are among the most radiosensitive plants, whereas *A. thaliana* and grasses are far more radiotolerant (Caplin and Willey 2018; Blagojevic et al. 2019). The reasons for the differential sensitivity have remained elusive. However, a recent comparative study showed that Norway spruce seedlings have a weaker and slower mobilization of genes involved in antioxidant protection, DNA repair, and stress tolerance than *A. thaliana* after exposure to 48 h of gamma radiation (Bhattacharjee et al. 2023). On the other hand, Norway spruce showed far more regulation of epigenetically related genes at high gamma dose rates, including strong upregulation of the DNA methylation machinery, a mechanism suggested to stabilize the genome (Bhattacharjee et al. 2023). This is consistent with the global hypermethylation observed in Scots pine trees growing in areas near Chernobyl with high levels of ionizing radiation (Kovalchuk et al. 2003; Volkova et al. 2018). A later study confirmed a dose-dependent hypermethylation of Scots pine in the Chernobyl accident zone, indicating a long-term epigenetic effect of ionizing radiation (Bondarenko et al. 2023). By contrast, Japanese red pine (*Pinus densiflora*) from areas affected by the 2011 Fukushima nuclear power plant accident showed increased hypomethylation (Bondarenko et al. 2023). These contrasting results suggest that there are differences between species and/or an environmental impact on epigenetic responses to radiation.

There is very little information about an epigenetic memory of ionizing radiation stress during seed development in conifers. Such a memory, and even trans-generational effects associated with DNA hypermethylation, have been observed in species such as *A. thaliana*, pea (*Pisum sativum*), and soybean (*Glycine max*) (Horemans et al. 2019). Interestingly, exposure to 144 h of gamma radiation caused less DNA damage in Scots pine seedlings grown from seeds that developed in Chernobyl areas with high and intermediate radiation levels than it did to seedlings from seeds in Chernobyl areas with background-level radiation (Rahman 2021). These studies suggest that epigenetic mechanisms are involved in a memory of ionizing radiation that is imprinted during embryogenesis.

## Biotic stress memory

The first evidence of biotic stress memory in conifers came from studies that used wounding and sublethal fungal inoculations to increase the resistance of Norway spruce trees to subsequent massive inoculation and bark beetle attack (Christiansen and Krokene 1999; Krokene et al. 1999). The most studied stimulus of biotic stress memory in conifers is the plant hormone methyl jasmonate (MeJA). MeJA has been used for more than 20 years to study defense priming and other inducible defenses in conifers (Martin et al. 2002; Hudgins et al. 2004).

**Figure 4.**
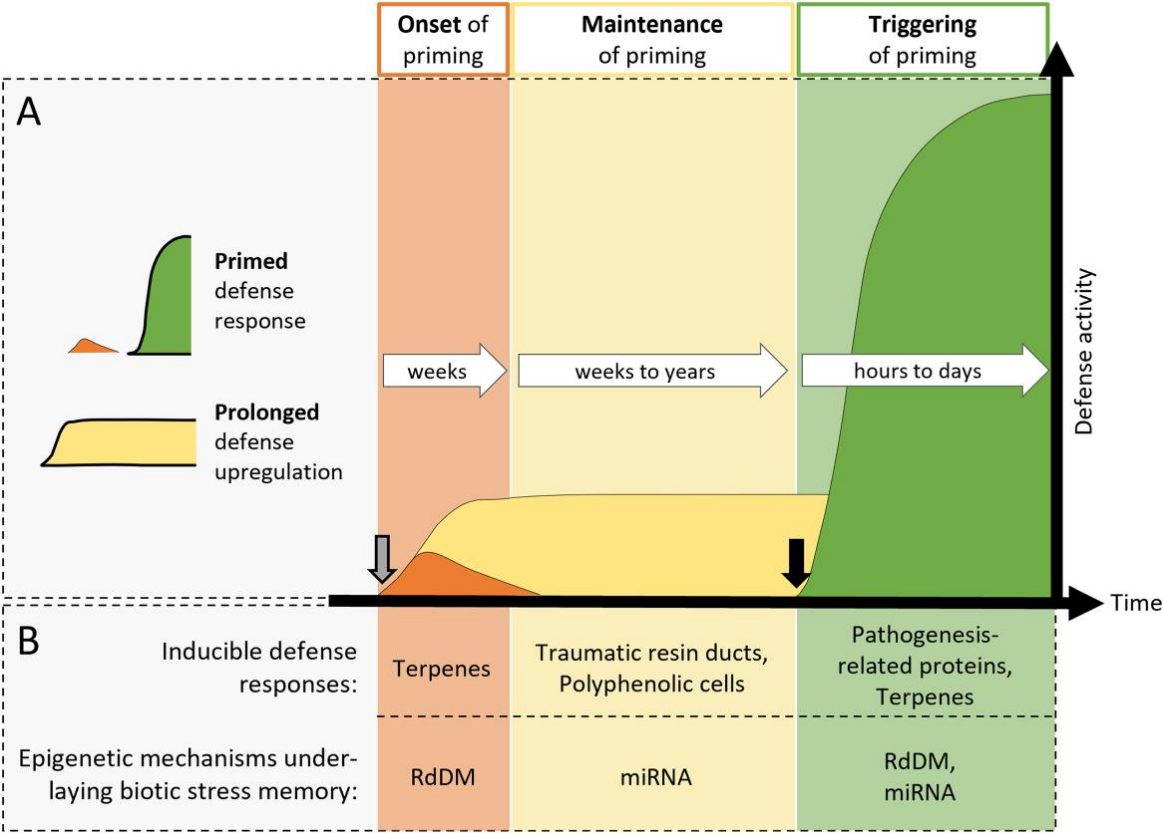
Biotic stress memory. **A)** Exposure of conifers to biotic stress or other stimuli (grey arrow), such as methyl jasmonate (MeJA) application, induces complex defense responses that include primed defenses and prolonged upregulated defenses. Primed defenses are latent until a trigger stress (black arrow) results in a strong and rapid induction. **B)** Conifer inducible defense responses include the accumulation of defensive molecules, formation of specialized defense structures, and synthesis of defensive enzymes. These defenses are executed in different tissues and over different time scales. Epigenetic mechanisms underlaying biotic stress memory likely include changes in DNA methylation, as many components of the RNA-directed DNA methylation (RdDM) pathway are temporarily downregulated during the onset of priming and after a trigger stress. Additionally, miRNAs that are differentially expressed may help maintain the primed state and the primed defense response by post-transcriptional silencing of mRNAs or guiding DNA methylation via the RdDM pathway.

Conifers have complex inducible defense responses that include direct induction, prolonged upregulation, and priming of defenses (Fig. 4) (Franceschi et al. 2005; Mageroy et al. 2020b). Directly induced defenses are activated immediately after attack or infection but return to near basal levels after a few days, whereas prolonged upregulated defenses remain activated for weeks to months. Primed defenses are a form of latent defense induction. Priming may involve a mild, temporary upregulation of induced defenses before defense activities return to basal levels (Wilkinson et al. 2022). In Norway spruce, primed defenses can remain latent for months to years until they are triggered by a subsequent stimulus, such as insect attack (Mageroy et al. 2020a; Chen et al. 2021; Krokene et al. 2023b) (Fig. 4). Triggering of priming involves a faster and/or stronger induction of inducible defenses compared to the response of a naïve, unprimed plant. Only recently has it been shown that MeJA directly induces, prolongedly upregulates, and primes defenses in Norway spruce (Mageroy et al. 2020a, b) (Fig. 4).

MeJA sensitizes some defense responses, such as synthesis of pathogenesis-related (PR) proteins, for stronger response to a trigger stress for at least 4 weeks after MeJA treatment (Mageroy et al. 2020b). The absolute duration of this MeJA-induced sensitivity is not known, but some studies indicate that it may last for years (Zas et al. 2014; Krokene et al. 2023b). As with temperature, hormones applied during embryogenesis have also been shown to have long-lasting effects on spruce resistance. This became apparent when Norway spruce saplings generated through somatic embryogenesis (SE) were challenged with herbivore attack (bark feeding by the large pine weevil *Hylobius abietis*) (Berggren et al. 2023). The study by Berggren and coworkers also suggests that SE and MeJA-treatment have a synergistic effect on reduced pine weevil feeding over a 3-year period (Berggren et al. 2023). In addition to plant defense hormones, insect sex hormones can also prime conifer defenses. Prior exposure of pine needles to sawfly sex hormones primes induced defenses to sawfly oviposition (Bittner et al. 2019).

Microbial symbionts can also induce biotic stress memory in conifers. For example, root colonization by ectomychorrizal fungi has been shown to provide protection against subsequent pathogen infection in pine (Wang et al. 2022; Chen et al. 2023). Priming of callose deposition may be involved in this ectomychorriza-induced resistance (Wang et al. 2022). This resembles the primed defense responses to *Botrytis cinerea* infection in tomato (*Solanum lycopesicum*) colonized by arbuscular mycorrhizae (Sanmartín et al. 2021).

Little is known about the precise epigenetic mechanisms involved in biotic stress memory in conifers. A recent time-course study of transcriptional changes in Norway spruce after MeJA treatment indicated that genes related to jasmonic acid (JA), salicylic acid (SA), ethylene biosynthesis, and downstream signaling pathways are temporarily upregulated (Wilkinson et al. 2022). Interestingly, the study found no evidence for the JA-SA defense hormone crosstalk that occurs in *A. thaliana* (Robert-Seilaniantz et al. 2011; Proietti et al. 2018; Aerts et al. 2021). Genes encoding the DNA methylation machinery were repressed, indicating that MeJA induces hypomethylation of the spruce genome (Wilkinson et al. 2022). Whole-genome bisulfite sequencing analysis is currently underway to determine where DNA methylation changes occur (Wilkinson, unpublished). Transcriptional analysis of MeJA-treated plants after wounding indicates that pathogenesis-related and cell wall-related genes show primed expression responses in Norway spruce (Mageroy et al. 2020b).

In addition to inducing transcriptional changes in mRNAs, MeJA application also augments the expression of miRNAs, which may be involved in post-transcriptional regulation and RNA-directed DNA methylation (Wilkinson et al. 2021). Many differentially expressed miRNAs in Norway spruce were predicted to target nucleotide-binding site leucine-rich repeat (NBS-LRR) transcripts (Wilkinson et al. 2021). This family of receptors is important for pathogen sensing and plant defense signaling and are highly expanded and diversified in conifers (Van Ghelder et al. 2019). *NBR-LRR*s also form the largest group of phased secondary small interfering RNA loci (PHAS loci) in the spruce genome (Xia et al. 2015). In monocots, phasiRNAs have been implicated in RdDM and may thus have a role in regulating epigenetic memory (Yu et al. 2021; Zhang et al. 2021).

## Conclusions/Future perspectives

Improving long-read sequencing technologies, assembly tools, and analysis pipelines will help us decipher the very large gymnosperm genomes, as has recently been demonstrated by Niu et al. (2022). This team used a combination of Illumina and PacBio sequencing of RNA and DNA to make a chromosome-level assembly and annotation of the largest gymnosperm genome so far (see Advances Box). This high-quality genome assembly clearly demonstrates that the large genome size of conifers is due to huge intergenic regions and long introns with a high content of transposable elements (TEs). Niu and co-workers also did whole-genome bisulfite sequencing and showed that TE-containing genes were much more highly methylated than TE-barren genes. This raises interesting questions about the role of TEs in regulating epigenetic memories in conifers (see Outstanding Questions Box). In addition to helping us better understand TEs, high-quality genomes and the biocomputing tools developed to produce them will facilitate the use of epigenetic methods in gymnosperms. Techniques that require whole-genome resequencing and peak-calling, such as chromatin immunoprecipitation sequencing (ChIP-Seq), have previously been too demanding to perform in these plants (Box 2).

**Figure 5.**
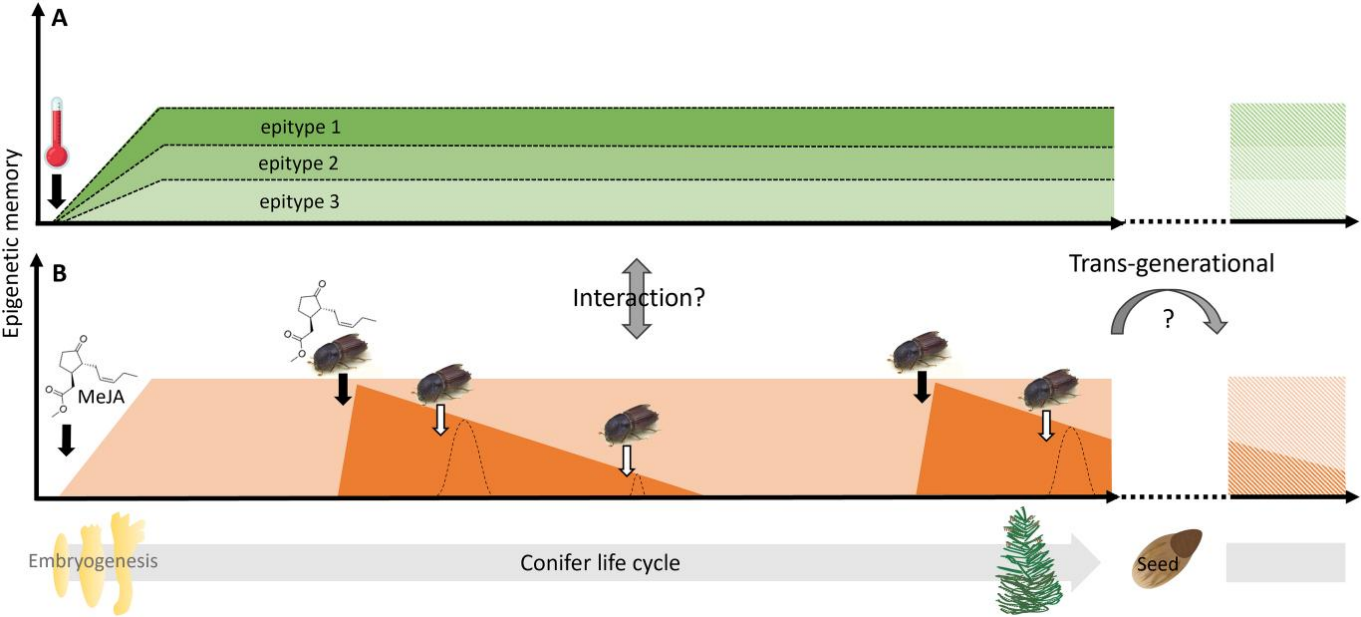
Induction, duration, and phenotypic manifestation of conifer epigenetic memory. Memory can be induced by various stimuli, such as temperature, phytohormones, and insect attack (black arrows). **A)** Climatic memory induced during embryogenesis is known to be very long-term (>20 years) (green forms). Different kinds of climatic stimuli may result in different epitypes being induced from a single genotype (epitype 1 to 3). These epitypes have different phenologies that may remain altered for the rest of the plants’ life. **B)** Biotic stress memory is manifested phenotypically in response to a triggering stimulus such as an insect attack (white arrows), seems to be attenuated over time (<4 years) (dark orange forms), and is manifested more short-term (bell-shaped curves) than climatic memory. There are still many unknowns about conifer epigenetic memory. For example, does induction during embryogenesis result in a more long-term memory resembling the climatic memory (light orange form)? Is epigenetic memory in conifers trans-generational (hashed green and orange forms)? Are there interactions between climatic and biotic stress memory?

Harnessing epigenetic memory is a promising approach to increase the adaptive potential of conifer trees (Yakovlev et al. 2012; Solvin and Steffenrem 2019). However, important knowledge gaps must be filled before epigenetics can be used effectively in silviculture. It is not well understood exactly how epigenetic memory is formed and how long it is maintained (Fig. 5). Very long-term epigenetic memory, lasting at least 20 years, has been demonstrated in Norway spruce trees in response to the temperature conditions experienced during embryogenesis. The longevity of biotic stress memory appears to be shorter. Some studies indicate that MeJA-induced memory increases tree resistance to pests and pathogens for 2 to 3 years, and other studies indicate that previous bark beetle attacks do not prime tree defenses to pathogen infection after 4 years (Zas et al. 2014; Nagel et al. 2022; Krokene et al. 2023b). Perhaps epigenetic memory induced by abiotic stress is more long-lasting than memory induced by biotic stresses (see Outstanding Questions Box). Alternatively, it could be that epigenetic memory established during embryogenesis is more long-lasting than memory established later in life (Fig. 5).

Another poorly known topic is how different stress memories interact with each other (Fig. 5). One recent article suggests that mild drought stress enhances spruce resistance to fungal infection, whereas severe drought makes trees more susceptible (Krokene et al. 2023a). Disentangling the interacting effects of abiotic and biotic stressors on epigenetic memory is difficult, as abiotic stressors often affect the performance of biotic stressors (e.g. when severe drought makes trees more susceptible to insect attack). With predicted increases in drought frequencies and biotic stress, we need more knowledge about interactions between abiotic and biotic stress memory in conifers (Hlásny et al. 2021; Machado Nunes Romeiro et al. 2022; Munro et al. 2022; Raffa et al. 2023).

It is still unknown if epigenetic marks can be passed from parent to offspring in gymnosperms. However, studies to investigate trans-generational transfer of the climatic memory in Norway spruce are underway (see Box 3 and the Outstanding Questions Box). It also remains to be shown if epigenetic traits that increase phenotypic plasticity show genetic variation and thus can be selected for. Furthermore, we still have limited understanding of epigenetic flexibility and allelic variation in epigenetic machinery genes in gymnosperms. A genetic basis for phenotypic plasticity is indicated by the extensive variation in epigenetic memory observed among Norway spruce families (Johnsen et al. 2005b; Yakovlev et al. 2011). However, so far, no allelic variants have been identified for epigenetic machinery genes or quantitative trait loci associated with conifer epigenetic memory. Exploiting epigenetically controlled plasticity in forest breeding and management may be key to creating flexible and resilient forests that will tolerate rapid global change and increasing disease pressures.

**Advances box**

- Improved sequencing tools: new sequencing technologies and bioinformatic tools are now enabling chromosome-level assembly of highly complex and repetitive conifer genomes. This is nicely illustrated by the 2023 publication of the full genome and methylome sequence of Chinese pine (*Pinus tabuliformis*).
- Trans-generational studies: 20-year-old temperature epitypes of Norway spruce have reached sexual maturity and will enable the first studies of trans-generational transfer of epigenetic memory in conifers.
- Harnessing epigenetic flexibility in forestry: improvements in sequencing technologies are enabling epigenetic studies in conifers at the population level. This lays the foundation for exploiting phenotypic plasticity to improve productivity and climatic adaptation in forestry trees.

**Outstanding questions box**

- Do conifers have a non-conical RdDM pathway? Unlike angiosperms, conifers do not have 24 nt microRNAs in vegetative tissues. These short RNAs regulate DNA methylation in angiosperms. What are the mechanisms of RdDM in conifers and other gymnosperms?
- What is the role of transposable elements (TEs) in conifer epigenetics? Conifer genomes are greatly expanded due to ancient insertions of TEs. Are TEs involved in epigenetic regulation?
- How long does epigenetic memory last? The limited available evidence suggests a lifelong duration of abiotic temperature memory and much less (a few years) for biotic stress memory.
- Can epigenetic memory be inherited across generations? Do temperature and biotic stress memory differ in this respect, and what mechanisms are involved in trans-generational inheritance?
- Do memories of abiotic and biotic stresses interact and, if so, how?

## Data Availability

No new data were generated or analysed in support of this research.

## Dive Curated Terms

The following phenotypic, genotypic, and functional terms are of significance to the work described in this paper:

bud AmiGo: PO:0000055

tes Gramene: AT3G43210
tes Araport: AT3G43210

LD Gramene: AT4G02560
LD Araport: AT4G02560

NRPE1 Gramene: AT2G40030
NRPE1 Araport: AT2G40030

FLC Gramene: AT5G10140
FLC Araport: AT5G10140

MSI1 Gramene: AT5G58230
MSI1 Araport: AT5G58230
